# Small-Target Detection Algorithm Based on STDA-YOLOv8

**DOI:** 10.3390/s25092861

**Published:** 2025-04-30

**Authors:** Cun Li, Shuhai Jiang, Xunan Cao

**Affiliations:** School of Mechanical and Electronic Engineering, Nanjing Forestry University, Nanjing 210037, China; lc010130@163.com (C.L.); 18934515584@163.com (X.C.)

**Keywords:** small-target detection, contextual augmentation, feature refinement, YOLOv8

## Abstract

Due to the inherent limitations of detection networks and the imbalance in training data, small-target detection has always been a challenging issue in the field of target detection. To address the issues of false positives and missed detections in small-target detection scenarios, a new algorithm based on STDA-YOLOv8 is proposed for small-target detection. A novel network architecture for small-target detection is designed, incorporating a Contextual Augmentation Module (CAM) and a Feature Refinement Module (FRM) to enhance the detection performance for small targets. The CAM introduces multi-scale dilated convolutions, where convolutional kernels with different dilation rates capture contextual information from various receptive fields, enabling more accurate extraction of small-target features. The FRM performs adaptive feature fusion in both channel and spatial dimensions, significantly improving the detection precision for small targets. Addressing the issue of a significant disparity in the number of annotations between small and larger objects in existing classic public datasets, a new data augmentation method called Copy–Reduce–Paste is introduced. Ablation and comparative experiments conducted on the proposed STDA-YOLOv8 model demonstrate that on the VisDrone dataset, its accuracy improved by 5.3% compared to YOLOv8, reaching 93.5%; on the PASCAL VOC dataset, its accuracy increased by 5.7% compared to YOLOv8, achieving 94.2%, outperforming current mainstream target detection models and small-target detection algorithms like QueryDet, effectively enhancing small-target detection capabilities.

## 1. Introduction

Small-target detection is a critical research direction in the field of target detection, aimed at precisely identifying objects of interest in small-target scenarios and determining their categories, shapes, positions, and other information. This provides perceptual support for various applications such as obstacle avoidance, interaction, and motion adjustment. Small-target detection has broad applicability and can be utilized in multiple areas, including intelligent surveillance, robot navigation, industrial automation, etc. By capturing distant small object information, systems can gain longer decision-making time, thereby better avoiding potential risks [1]. Therefore, researching efficient small-target detection methods holds significant application value and research importance.

Since Ross G. et al. [2] introduced the use of regions with CNN features (RCNNs) for target detection, deep learning-based target detection algorithms have evolved. Deep learning target detection algorithms are divided into two-stage and single-stage algorithms. Common two-stage algorithms include R-CNN [3], SPPNet [4], Fast RCNN [5], Faster RCNN [6], Feature Pyramid Networks (FPNs) [7], etc., comprising two separate network models: a Region Proposal Network (RPN) that generates candidate bounding boxes and a network that classifies these boxes and performs bounding box regression. Two-stage algorithms utilize RPN for precise candidate box generation, reducing false positive rates and achieving higher accuracy; however, they require computation across two stages, making them slower. Thus, two-stage algorithms are suitable for large targets and complex scenes, leaving much room for improvement in detecting small objects. Common single-stage algorithms like YOLO [8], SSD [9], RetinaNet [10], etc., contain a single network model capable of simultaneously estimating object positions and classifying them, offering faster speeds. In recent years, the YOLOv8 algorithm has been applied in the target-detection field due to its high accuracy and real-time detection advantages, yet it still exhibits high false positive and miss rates in small-target localization and detection.

The poor performance of small-target detection is mainly due to the inherent limitations of networks and imbalanced training data [11]. To obtain accurate semantic information, new detection algorithms incorporate increasing numbers of pooling and downsampling operations, causing the gradual loss of features from small objects with fewer pixels during iterations, which limits the detection performance of small targets [12]. FPN connects low-resolution, high-semantic information from higher layers and high-resolution, low-semantic information from lower layers through a top-down lateral connection, enriching the semantic information at all scales and alleviating the issue of information loss to some extent. Zhang F. et al. [13] introduced a high-resolution attention mechanism into FPN to mine small-target information. PANet [14] added a bottom-up fusion path based on FPN, allowing effective information transfer from lower to upper layers. ASFF [15] adaptively learns spatial weights at different scales, addressing the inconsistency of candidate boxes across different scale feature maps. NAS-FPN [16] and BiFPN [17] search for an effective block within FPN, repeatedly stacking it to elastically control the FPN size and improve efficiency. These structures significantly enhance the multi-scale representation capability of the network, but directly fusing information from different scales can cause semantic conflicts, limiting multi-scale feature expression and submerging small object information amidst conflicting information. Additionally, in existing classic public datasets, the number of small target annotations is far less than that of larger targets [18]. During training, the network continuously converges towards larger targets, leading to poor performance on small targets.

To address the issues of network limitations and imbalanced training data in small-target detection, this paper proposes an improved YOLOv8 small-target detection algorithm, STDA-YOLOv8 (small-target detection algorithm based on YOLOv8), which enhances the detection accuracy of small targets while ensuring real-time detection. The main contributions of this paper are as follows:

(1) The STDA-YOLOv8 algorithm is proposed to significantly improve the performance of small-target detection by combining the Context Augmentation Module (CAM) and Feature Refinement Module (FRM).
(2) A new data enhancement method (Copy–Reduce–Paste) is proposed, which effectively solves the problem of an insufficient number of small targets in the training data and makes the training process more balanced.
(3) The experiments show that the mAP of the STDA-YOLOv8 model on the VisDrone dataset and the PASCAL VOC dataset reaches 93.5% and 94.2%, respectively, which is 5.3% and 5.7% higher, respectively, than that of the original YOLOv8, which reflects a significant performance advantage, and there is no significant increase in the model complexity.

## 2. Related Work

### 2.1. Target Detection Based on Deep Learning

The main purpose of target detection is to identify and locate objects in images or videos, which can be considered a regression problem. Before the advent of deep learning, traditional target detection algorithms relied on hand-crafted features, which had weak generalization capabilities and performed poorly in complex scenes.

In 2014, the introduction of the two-stage target detection algorithm R-CNN [3] ushered in the era of deep learning for target detection. In 2015, Joseph Redmon and others proposed YOLOv1 [8], which predicts the position and category of objects in an image through single forward propagation. YOLOv2 [19] improved upon YOLOv1 by introducing batch normalization, using high-resolution images for training, and adopting a fully convolutional network structure. YOLOv3 [20] employed a deeper Darknet-53 as its backbone and introduced FPN to enhance the detection ability for objects of different scales. To further improve speed and accuracy, Alexey Bochkovskiy and others proposed YOLOv4 [21], incorporating advanced techniques such as CSPNet, PANet, and SAM for more efficient feature extraction and target detection. YOLOv5 further enhanced the algorithm’s speed and accuracy while simplifying the code structure for easier use and deployment.

Wang J. et al. [22] proposed an attention-improved YOLOv5 target detection algorithm, UTD-Yolov5, which replaces the original backbone with a two-stage cascaded CSP and introduces a visual channel attention mechanism module SE. It also designed a random anchor similarity calculation method, WBF, and iterative refinement mechanisms, enabling more flexible detection and accurate feature capture. Feng Jiangfan et al. [23] presented a lightweight solution that includes a cross-stage partial bottleneck transformer (CSP BoT) module and an angular classification prediction branch. They also combined the prediction head network with an adaptive spatial feature fusion block (ASFF-Head) to further improve multi-scale feature maps, accommodating spatial variations in prediction uncertainty. Chunshan W. et al. [24] modified the BottleNeck structure to enhance the model’s multi-scale feature capture capability, embedded the SimAM attention mechanism module to focus on key features without increasing parameter count, and added Vision Transformer components to improve global feature perception within images. Zhai Xianyi et al. [25] introduced a Multi-Scale Retinex with Color Restoration (MSRCR) algorithm for image processing to enhance contrast, added a Convolutional Block Attention Module (CBAM) to improve recognition precision and efficiency, and incorporated a detect layer into the YOLOv5s head network to precisely identify small targets. Jiangjie X. et al. [26] integrated an OCAM (Object Convolution Attention Module) into the feature extraction layer of the YOLOv5 network. This module builds contextual relationships between features to enhance their relevance, establishing long-range dependencies between channel features and positional features, effectively improving detection performance.

YOLOv6 introduced the new RepVGG architecture with an anchor-free detector, enhancing the model’s adaptability for various industrial applications. YOLOv7 proposed several architectural changes and a series of free packages, optimizing model structure re-parameterization and dynamic label assignment issues to improve accuracy while maintaining real-time performance. YOLOv8 introduced new features and improvements to further enhance performance and flexibility, including a new backbone network, a new anchor-free detection head, and a new loss function, aiding in higher detection accuracy suitable for real-time target detection tasks in diverse application fields [27]. Recent studies such as Ashraf et al. [28] analyzed the performance of the YOLO series of models under degraded imaging conditions, and Varghese et al. [29] and Chai et al. [30] proposed an improved version of YOLOv8, which was optimized for model performance and small-target detection, respectively, and further promoted the application of the YOLO algorithm to real complex scenes.

As a landmark framework in the field of deep learning-based target detection, the YOLO series has continuously evolved to meet the complex and diverse demands of detection tasks [31]. However, due to the small pixel size of small objects, extracting effective information is challenging, posing significant difficulties and challenges for small-target detection. Among the current YOLO versions, YOLOv8 better maintains detection accuracy while improving detection speed. Therefore, this paper adopts YOLOv8 as the baseline framework.

### 2.2. Multi-Scale Feature Fusion

Multi-scale feature fusion enhances the understanding of image and video content by capturing feature information at different scales and levels, providing a solid foundation for applications in image processing, computer vision, and deep learning.

MSGNet [32] leverages frequency domain analysis and adaptive graph convolution to capture inter-series correlations across multiple temporal scales, demonstrating good generalization capabilities. CEDNet [33], with its simplified cascade encoder–decoder network designed for dense prediction tasks, performs multi-scale feature fusion in the decoder. The DAU-FI Net architecture [34], primarily aimed at semantic segmentation of imbalanced datasets, integrates a multi-scale spatial channel attention mechanism and feature injection to improve accuracy. Its core consists of a multi-scale depthwise separable convolution block and a spatial-channel compression and excitation (scSE) attention unit, modeling channel and spatial region dependencies within feature maps. DAU-FI Net utilizes additive attention gates to optimize segmentation and expand the feature space. The Multi-Scale Dilated Attention (MSDA) module [35] models local and sparse patch interactions, constructing a multi-scale dilated transformer (DilateFormer) that excels in visual tasks. The Concentrated Feature Pyramid (CFP) [36] optimizes features through global explicit supervision. CFP employs a lightweight MLP to capture global dependencies and focuses on image corner regions through a learnable visual center mechanism. This method adjusts shallow features using information extracted from deeper layers, achieving a more comprehensive and distinctive feature representation that enhances the performance of YOLOv5 and YOLOX baselines in target detection.

Despite significant progress in target detection through multi-scale feature fusion methods, there are still limitations in small-target detection tasks. For example, the FPN structure [7], which fuses high-level semantic information into low-resolution features through a top-down approach, lacks rich contextual information for small objects during processing, leading to insufficient expression of small object features and limited detection performance. Although PANet [14] improves bidirectional feature flow through a bottom-up pathway, semantic inconsistencies between features of different scales persist, especially as small objects can be overwhelmed by stronger high-level semantic information.

To address these issues, this paper proposes a Contextual Augmentation Module (CAM) and a Feature Refinement Module (FRM). The CAM captures contextual information from different receptive fields through multi-scale dilated convolution, compensating for the FPN’s shortcomings in acquiring contextual information, particularly suitable for extracting small-object features. The FRM resolves conflicts between features of different scales by adaptively fusing features along channel and spatial dimensions, ensuring that small-object features are not overshadowed during fusion. Consequently, our method effectively improves the accuracy and reliability of small-target detection.

## 3. Methodology

### 3.1. YOLOv8 Model

YOLOv8 builds upon the foundational network architecture concepts of the YOLO series, introducing various improvements and innovations. Its network structure primarily consists of three components: the backbone, neck, and head [29].

The backbone serves as the foundation of the model, adopting a structure similar to CSPDarknet, which includes the CBS module, C2f module, and SPPF module, responsible for feature extraction from input images. The C2f module ensures that YOLOv8 remains lightweight while capturing richer gradient flow information. The SPPF module replaces the SPP module in YOLOv5, reducing execution time by half while retaining the functionality of transforming feature maps of any size into fixed-size feature vectors.

**Figure 1:**
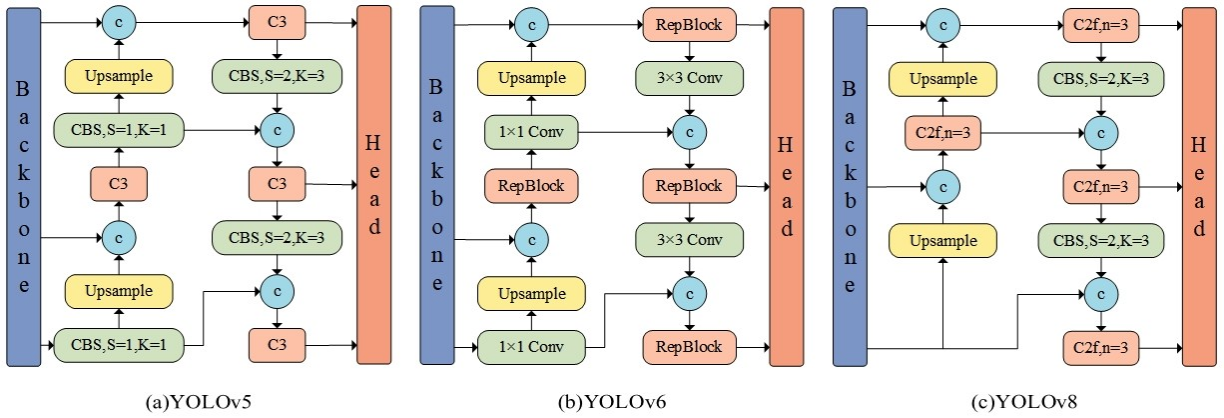
The neck structures of various YOLO algorithms.

The neck, positioned between the backbone and head, is composed of FPN and PAN structures, tasked with feature fusion and enhancement. As illustrated in Figure 1, compared to YOLOv5 and YOLOv6, YOLOv8 replaces the C3 module and RepBlock module with C2f and removes the 1 × 1 convolution before upsampling, directly feeding features from different stages of the backbone into the upsampling operation.

The detection head is the decision-making component of target detection, employing a decoupled head approach that separately extracts target position and category information through different network branches. These branches are trained independently before fusion to produce the final detection results. This method effectively reduces the number of parameters and computational complexity, enhancing the model’s generalization capability and robustness.

### 3.2. YOLOv8 Model Enhancements

**Figure 2:**
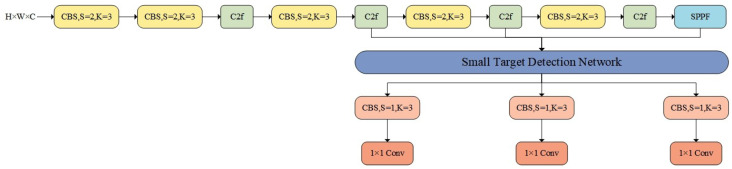
STDA-YOLOv8 general structure.

Applying the YOLOv8 model to small-target detection in the field of autonomous driving still yields relatively poor performance on road surfaces [30]. To address this issue, this paper introduces a new small-target detection network structure—STDA-YOLOv8, which is integrated into the fundamental framework of YOLOv8, as shown in Figure 2.

**Figure 3:**
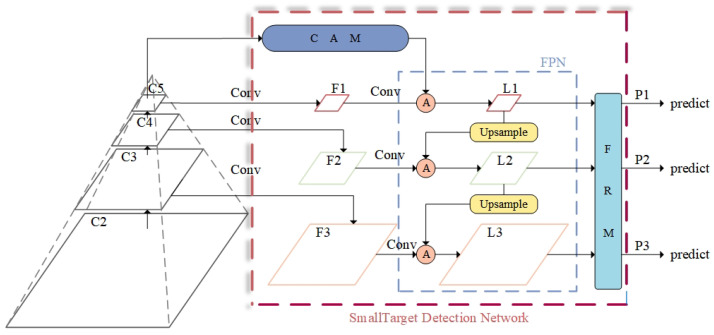
Structure of small-target detection network.

This includes a new small-target detection network structure, as shown in Figure 3. This structure combines the Contextual Augmentation Module and the Feature Refinement Module, enhancing small-target detection performance through multi-scale dilated convolution and adaptive feature fusion.

The CAM enhances the contextual information needed for small-target detection through multi-scale dilated convolution. Compared to the traditional FPN, the CAM obtains richer contextual information using convolutional kernels with different dilation rates, especially effective at capturing detailed features of small objects at low resolutions. This improves the model’s detection of small objects without significantly increasing computational complexity.

The FRM addresses conflicts between features of different scales by adaptively fusing features along both channel and spatial dimensions. Compared to attention mechanisms like the SE module, the FRM better preserves the effective information of multi-scale features, particularly preventing small-object features from being overshadowed during fusion. This innovative design makes the FRM more suitable forsmall-target detection tasks, significantly improving detection accuracy.

#### 3.2.1. CAM Module

**Figure 4:**
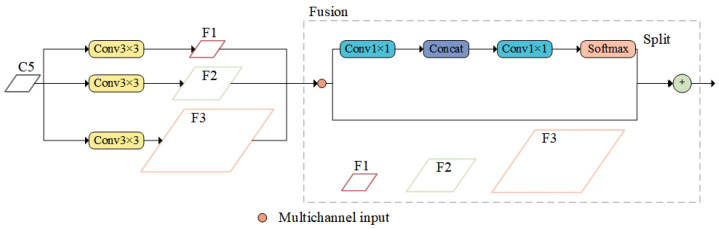
CAM structure.

In order to enrich the contextual information required for small-target detection, the CAM (Contextual Augmentation Module) is proposed, where “A” stands for augmentation, which reflects the core idea of the module to improve the detection performance by enhancing the contextual information of small targets. The CAM is applied to the neck structure of YOLOv8, where different scale feature maps (C3, C4, C5, respectively) extracted from the backbone network are used as inputs to the CAM in order to generate augmented multi-scale feature maps F1, F2, F3 (corresponding to different scale levels). Figure 4 shows the specific module structure of the CAM as an example when inputting the feature maps of layer C5 of the backbone network, which supplements the contextual information required for small-target detection by introducing multi-scale expansion convolution, using convolution kernels with different dilation rates to obtain contextual information from different receptive fields and injecting this information into the various layers of the FPN.

Dilation convolution, also known as null convolution, expands the convolution’s sense field by introducing intervals (dilation rates) in the convolution kernel without increasing the number of parameters or computational complexity. The specific calculation formula is:

(1) $$\begin{array}{c} y\left(i,j\right)=\sum_{m=0}^{k-1}\left[\sum_{n=0}^{k-1}\left[x\left(i+r\cdot m,j+r\cdot n\right)\cdot w\left(m,n\right)\right)\right]] \end{array}$$

where *r* is the dilation rate and *k* is the convolution kernel size.

The advantage of dilated convolution is to increase the sensory field to obtain more contextual information without losing spatial resolution, which is especially important for capturing detailed features of small targets. Since small targets have fewer pixels and are easily ignored, we can efficiently extract rich contextual information from different spatial scales through multi-scale dilated convolution to enhance the recognition of small targets.

Taking convolutional kernel dilation rates of 1, 3, and 5 as examples, the formula for obtaining contextual information through multi-scale dilated convolution is as follows, and the calculation for other dilation rates follows the same principle:

(2) $$\begin{array}{c} CAM_{output}=Conv_{3*3}\left(Rate=1\right)+Conv_{3*3}\left(Rate=3\right)+Conv_{3*3}\left(Rate=5\right) \end{array}$$

where *Rate* represents the dilation rate, and Formula (2) indicates that multi-scale contextual information is acquired through convolution kernels with different dilation rates and then performs weighted fusion of this information.

**Figure 5:**
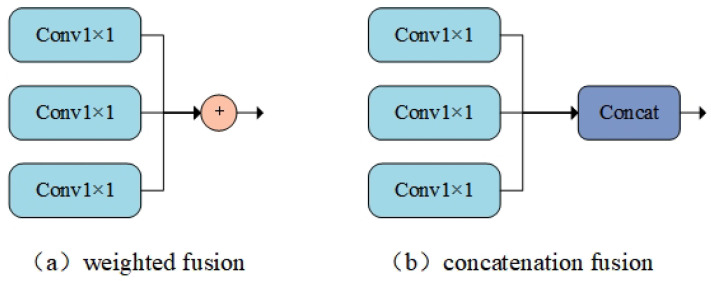
Feature fusion method.

Common feature fusion methods include weighted fusion and concatenation fusion, as shown in Figure 5.

Figure 5a,b represent weighted fusion and concatenation fusion, respectively, adding feature maps directly on the spatial and channel dimensions. Concatenation fusion connects two features directly, concatenating them along the input feature’s dimensionality. Weighted fusion employs a parallel strategy, combining two feature vectors into a complex vector. The formula for the weighted fusion method is as follows:

(3) $$Fusion_{weighted}=\sum_{i=1}^{n}\alpha_{i}+F_{i}$$

where $\alpha_{i}$ is the weighting coefficient and $F_{i}$ is the feature map at different scales.

In Figure 4, the fusion module employs an adaptive fusion method. Assuming the input size is (N, C, H, W), through convolution, fully connected layers, and Softmax operations, it obtains spatially adaptive weights of size (N, 3, H, W). The three channels correspond to the three inputs, and contextual information is integrated into the output by calculating a weighted sum. The formula for the adaptive fusion method is as follows:

(4) $$\begin{array}{c} Fusion_{adaptive}=Softmax\left(Conv_{1*1}\left(Concat\left(F_{1},F_{2},F_{3}\right)\right)\right) \end{array}$$

(5) $$Softmax\left(x\right)=exp\left(x_{i}\right)/\sum_{i}exp\left(x_{i}\right)$$

where *Concat* denotes the concatenation of multi-scale feature maps, $Conv_{1*1}$ denotes dimensionality reduction by 1∗1 convolution, and *Softmax* ensures that smaller values have lower probabilities and are not directly discarded, generating adaptive fusion weights.

#### 3.2.2. FRM

**Figure 6:**
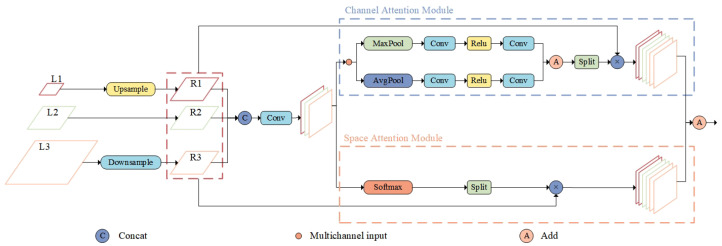
FRM structure.

To address the issue of redundant and conflicting information arising from direct fusion of features at different scales, which can reduce multi-scale expression capabilities, this paper introduces a Feature Refinement Module. Figure 6 shows the feature maps at different levels within the FRM. The FRM performs adaptive feature fusion along both channel and spatial dimensions, eliminating conflicting information between features of different scales to prevent small-object features from being overshadowed. This significantly enhances the fusion effect, ensuring that the feature information of small targets is preserved and reinforced, substantially improving the detection accuracy for small objects.

The FRM mainly consists of two branch modules, namely the Channel Attention Module and the Spatial Attention Module, which generate adaptive weights along the channel and spatial dimensions, respectively, enabling the network to focus on more important information. The channel attention mechanism emphasizes the relationships between channels by applying weights across them, helping the network learn the inter-channel relationships and extract significant channel information. Specifically, the input feature map is first compressed along the spatial dimension to obtain a spatial information representation containing global features of the image. Then, accurate global features are obtained through adaptive average pooling and adaptive max pooling. The formula for generating channel attention is as follows:

(6) $$K_{m}^{x,y}=a_{m}\cdot X_{\left(1,m\right)}^{x,y}+b_{m}\cdot X_{\left(2,m\right)}^{x,y}+c_{m}\cdot X_{\left(3,m\right)}^{x,y}$$

(7) $$\left[a_{m},b_{m},c_{m}\right]=\sigma \left[AP\left(F\right)+MP\left(F\right)\right]$$

(8) σ(x)=1/(1+exp(−x))

where $K_{m}^{x,y}$ denotes the output vector of the $m_{th}$ layer at position (x,y); *F* denotes the features generated by the concatenation operation; $a_{m},b_{m},c_{m}$ denotes the adaptive weights of size 1∗1∗1, which are generated by average pooling (*AP*) and maximum pooling (*MP*) of the feature maps followed by the σ(sigmoid) operation.

The Spatial Attention Module operates on the spatial dimension of the feature map, capturing spatial information at different scales to extract relevant spatial locations. This is achieved by generating relative weights for each location across channels using Softmax. The formula for generating spatial attention is as follows:

(9) $$\begin{array}{c} \phi_{m}^{x,y}=\sum_{c=1}^{3}\sum_{k,x,y}\left(\mu_{m}^{c,x,y}\cdot X_{\left(1,m\right)}^{k,x,y}+\upsilon_{m}^{c,x,y}\cdot X_{\left(2,m\right)}^{k,x,y}+\eta_{m}^{c,x,y}\cdot X_{\left(3,m\right)}^{k,x,y}\right) \end{array}$$

(10) $$\left[\mu_{m},\upsilon_{m},\eta_{m}\right]=Softmax\left(F\right)$$

where $\phi_{m}^{x,y}$ denotes the output vector of the $m_{th}$ layer at position (x,y) and $\mu_{m}^{c,x,y}$,$\upsilon_{m}^{c,x,y}$,$\eta_{m}^{c,x,y}$ denote the spatial attention weight relative to the $m_{th}$ layer, which is obtained by normalizing the feature maps using Softmax.

In summary, the generation formula for the total output of the FRM is as follows:

(11) $$p_{m}=K_{m}+\phi_{m}$$

The features of each layer of the FRM are fused together under the guidance of adaptive weights, and the final output of the whole network is $p_{1},p_{2},p_{3}$.

To summarize:

(1) Unlike traditional FPN and PANet, the CAM proposed in this paper effectively solves the problem of insufficient capturing of small-target contexts by traditional structures through multi-scale expansion convolution;
(2) The FRM performs adaptive fusion in both channel and spatial dimensions, which solves the problem of scale feature conflict in traditional feature fusion methods;
(3) The theoretical contribution lies not only in the innovation of module structure but also in the proposed data enhancement method (Copy–Reduce–Paste) which effectively solves the training data imbalance problem with clear theoretical and practical application implications.

## 4. Results and Analysis

### 4.1. Experimental Dataset and Experimental Setup

#### 4.1.1. Experimental Dataset

Experiments were conducted on the VisDrone dataset. The VisDrone dataset consists of 288 video segments, 261,908 frame images, and 10,209 static images, covering multiple aspects including locations (14 different cities in China), environments (urban and rural), objects (pedestrians, vehicles, bicycles, etc.), and densities (sparse and crowded scenes). It includes manually annotated bounding boxes for over 2.6 million targets such as pedestrians, cars, bicycles, and tricycles. To validate the applicability of STDA-YOLOv8, we also tested it on the PASCAL VOC dataset to ensure its effectiveness in small-target detection. These datasets were divided into training and testing sets at a ratio of 7:3. The training set was used to train the model, while the test set was used to detect the model’s detection accuracy and evaluate its generalization ability.

#### 4.1.2. Data Enhancement Method: Copy–Reduce–Paste (CRP)

In existing classic public datasets, the number of small-target annotations is significantly less than that of larger targets, causing the network to continuously converge towards larger targets during training and resulting in poor performance on small targets. To address this issue, this paper proposes a novel data augmentation method, called “Copy–Reduce–Paste” data augmentation. This method involves copying larger targets, shrinking their size, and then pasting them back into the original image to increase the contribution of small objects to the loss function during training, ensuring balanced training. In this study, a bounding box area larger than 64 × 64 pixels (corresponding to the input image size of 640 × 640 pixels) is defined as a ‘larger’ object, and a bounding box area smaller than 32 × 32 pixels is defined as a ‘small’ object.

For specific implementation, this paper first identifies larger targets with an area larger than 64 × 64 pixels in the original image, copies these targets and reduces them by a random proportion (usually 30% to 50% of the original size), and then randomly selects a blank area in the image for pasting. In order to prevent the pasted targets from overlapping with the existing targets, this paper implements a simple verification step: if there is any intersection between the randomly selected location and the location of the existing targets in the original image, a new blank location is randomly selected again to be pasted until the no-overlap condition is satisfied.

To further validate the effectiveness of this data augmentation method, we conducted a comparative analysis of the datasets before and after enhancement and evaluated the number of small targets and model performance under different scene types in detail. Table 1 shows the changes in the number of small targets and model performance metrics such as precision, recall, and mean average precision (mAP) before and after data augmentation on the VisDrone and PASCAL VOC datasets across different scenes, where ‘Quantity’ represents the quantity of small targets, ‘Proportion’ represents the proportion of small targets and large targets, ‘before’ indicates the situation before data augmentation, and ‘after’ indicates the situation after data augmentation.

From the table, it is evident that data augmentation effectively increased the proportion of small targets in the training data, significantly improving the model’s precision, recall, and mAP across different scenes. The performance enhancement was particularly notable in sparse and crowded scenarios.

#### 4.1.3. Experimental Setup

To ensure the accuracy of the experiments, all experiments were conducted on a Windows 11 operating system. The GPU platform used was an NVIDIA RTX 2060, and the hardware configuration remained consistent. The deep learning framework employed was Pytorch 2.2.2, with CUDA version 12.1. The programming language used was Python, version 3.9.19. Specific training parameter settings are detailed in Table 2.

### 4.2. Evaluation Metrics

Precision (*P*), recall (*R*) and mean average precision (*mAP*) are selected as the metrics to evaluate the model’s detection performance.

Precision measures the proportion of correctly predicted positive samples out of the total number of samples, while recall measures the proportion of correctly predicted positive samples out of the total labeled samples. The formulas are as follows:

(12) P=TP/(TP+FP)

(13) R=TP/(TP+FN)

where *TP* (true positives), *FP* (false positives) and *FN* (false negatives) represent the number of correctly detected targets, falsely detected backgrounds, and falsely detected targets as backgrounds, respectively.

Mean average precision takes into account the average precision (*AP*) of each category as well as the number of categories (*C*). The formula is as follows:

(14) $$mAP=\sum_{i=1}^{C}AP_{i}/C$$

### 4.3. Results and Comparison

#### 4.3.1. Ablation Study

To verify the effectiveness of each module in the model, this paper conducted ablation studies by adding modules one by one. Based on the original YOLOv8 model, we incorporated the CAM and the FRM. P, R, mAP, parameters, floating-point operations, and model size were used as evaluation metrics. The results of the ablation experiments are shown in Table 3 below.

It can be observed that the modules designed in this paper have improved the model’s detection performance for small targets to a certain extent. Building on the foundation of YOLOv8, adding CAMs with different dilation rates allows the model to capture features at various scales and levels, thereby enhancing the detection performance for small targets. Using smaller dilation rates (such as [1, 2, 3]) can maintain the precision of details and improve the detection rate of small objects, especially when the background is simple; using larger dilation rates (such as [2, 4, 6]) enhances the recognition ability of small targets in more complex backgrounds by increasing the coverage of the receptive field. The combination experiments of different dilation rates comprehensively demonstrate the effectiveness and flexibility of the CAM and its performance changes under different configurations, optimizing the model to find the best configuration for small-target-detection scenarios.

On top of adding the CAM, further improvements were made to YOLOv8 with the FRM, resulting in further enhancements in precision, recall, and $mAP_{0.5}$. Additionally, both the number of parameters and computational load decreased, indicating that the FRM not only reduced the model’s computational complexity and parameter count but also strengthened the learning capability of multi-scale features to some extent.

In some experimental data, while the number of parameters decreased, the model size increased. This could be due to reducing the parameter count while introducing non-parameterized structures or modules, leading to an increase in overall storage requirements for the model. For example, adding the FRM on top of CAMs with dilation rates of [1, 3, 5] reduced the parameter count, but this module might include more feature processing operations that require storing additional information within the model, slightly increasing the overall model size. Therefore, when improving and optimizing a model, it is essential to consider the specific impacts of each module on the number of parameters and model size to achieve optimal performance and resource utilization.

The experimental results show that when introducing three sets of CAMs with different dilation rates along with the FRM, the resulting STDA-YOLOv8 model achieved a 15.5% improvement in precision, a 12.6% improvement in recall, and a 6% improvement in $mAP_{0.5}$ compared to YOLOv8 on the VisDrone dataset; on the PASCAL VOC dataset, there was a 11.5% improvement in precision, a 10.1% improvement in recall, and a 6.4% improvement in $mAP_{0.5}$. Additionally, both the model’s parameter count and size were smaller than those of the baseline model, mainly due to the synergistic effect of the two modules. By reducing redundant features and optimizing multi-scale feature fusion, the complexity of the model was reduced. This optimization not only reduced the computational load but also decreased the model’s storage footprint, making the model more efficient.

In summary, the proposed STDA-YOLOv8 algorithm exhibits outstanding performance in small-target detection.

#### 4.3.2. Comparative Experiment

In order to evaluate the detection effect of the improved model, performance comparison experiments are conducted between STDA-YOLOv8 and the mainstream target detection models YOLOv5, YOLOv7, YOLOv8, the small-target detection algorithm QueryDet, and the target detection algorithms in the studies [37,38].The experiments are conducted using the same equipment, environment, and dataset for training and testing, and the results of the comparison experiments are shown in Table 4.

As can be seen from Table 4, the improved STDA-YOLOv8 model has an mAP metric of 93.5% on the VisDrone dataset, which is a 5.3% improvement compared to the original YOLOv8; a 10.5% improvement in recall; and a 12% improvement in precision. On the PASCAL VOC dataset, the mAP metric of STDA-YOLOv8 is 94.2%, which improves 5.7% compared to YOLOv8, and it improves 8.5% in recall and 9.3% in precision. The above results fully demonstrate that STDA-YOLOv8 outperforms YOLOv5, YOLOv7, YOLOv8, QueryDet, and the recently proposed YOLO-Z [37] and One-Shot PCB [38] methods, demonstrating significant performance advantages in small-target detection tasks. In addition, the number of parameters of this paper’s method is only $2.6\times 10^{6}$, and the computational complexity is 7.5 $GFLOP_{s}$, which are lower than the above comparative models, reflecting that the algorithm effectively reduces the complexity and resource requirements of the model while improving the performance and takes into account the requirements of high efficiency and real time, which makes it suitable for a wider range of practical application scenarios.

**Figure 7:**
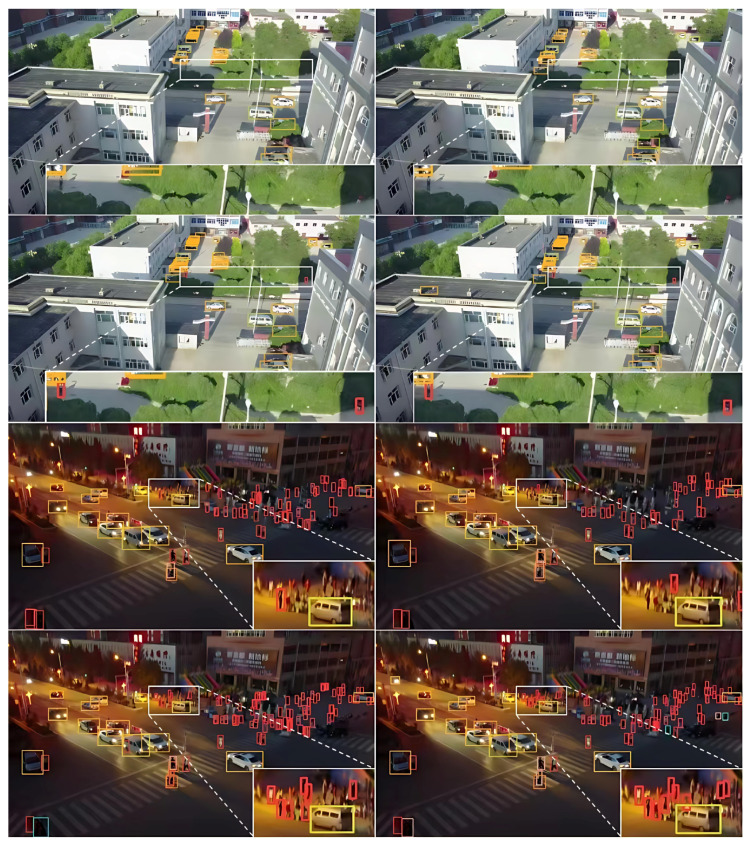
Plot of some of the detection results on the VisDrone dataset.

Figure 7 shows a comparison of some sample detection results on the VisDrone dataset. Among them, the original YOLOv8 algorithm is used in left side of figure, and it can be observed that there are obvious false positives, false negatives, and serious overlapping bounding boxes in the white magnified box region, and in right side of figure, these false positives, false negatives, and serious overlapping bounding boxes are significantly reduced and the overlapping bounding boxes are effectively improved in the same white zoomed-in box region, which shows that the proposed algorithm STDA-YOLOv8 has obvious advantages in the accuracy and robustness of small-target detection.

**Figure 8:**
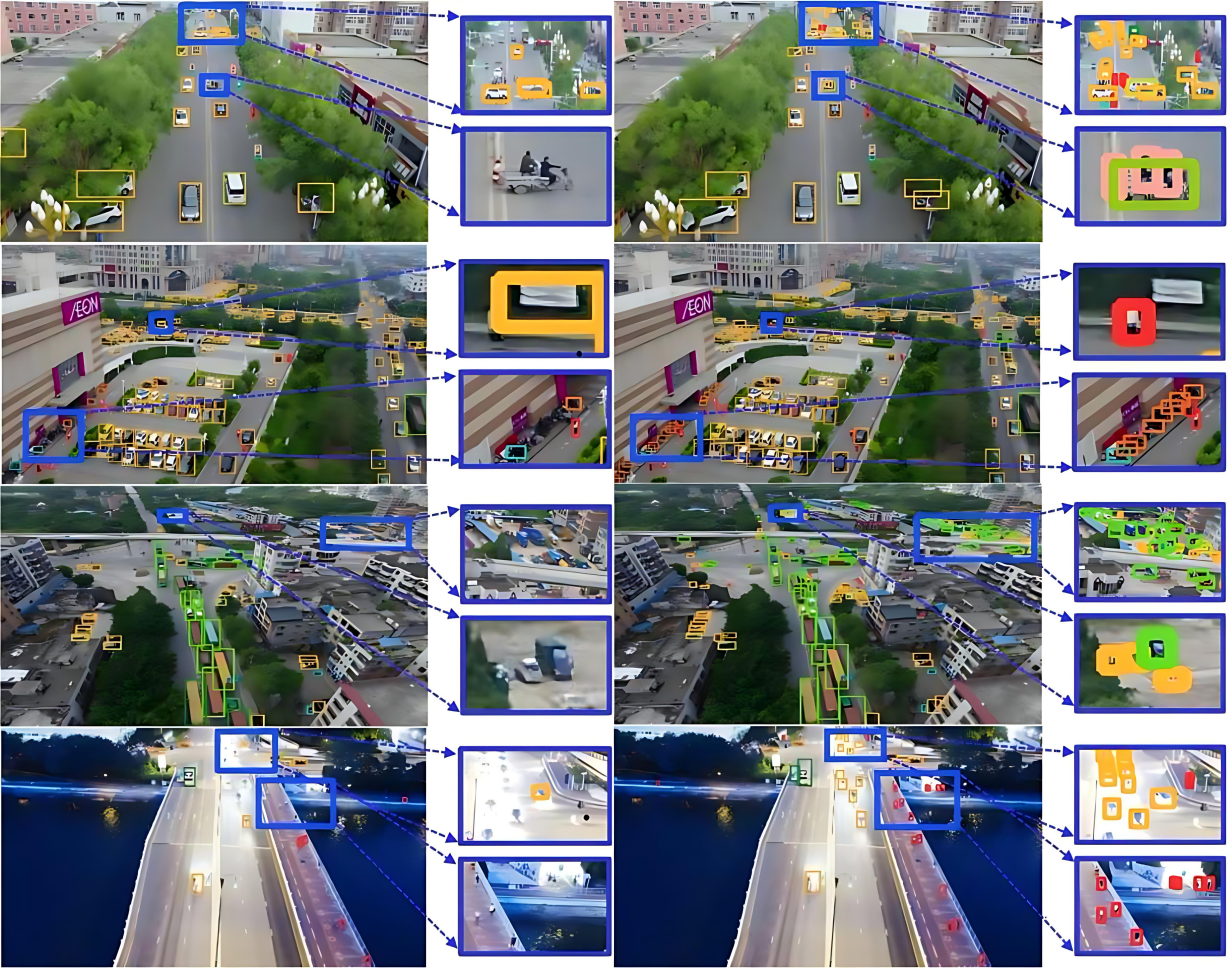
Plot of some of the detection results on the PASCAL VOC dataset.

To verify the applicability of STDA-YOLOv8, we also conducted testing on the PASCAL VOC dataset to ensure the algorithm’s effectiveness in small-target detection. Figure 8 shows a comparison of some sample detection results on the PASCAL VOC dataset. Among them, left side of Figure 8 shows the detection results of the original YOLOv8 algorithm, with multiple obvious misdetections and omissions in the blue zoomed-box region and insufficient recognition accuracy of small targets; right side of Figure 8 shows the detection results of the STDAYOLOv8 algorithm, with a significant reduction in misdetections and omissions in the same region and a significant increase in the detection accuracy and stability of small targets, which verifies the effectiveness of this paper’s method in a real environment.

#### 4.3.3. Evaluation of Computational Efficiency on Different Hardware Platforms

To comprehensively evaluate the computational efficiency of the proposed STDA-YOLOv8 model, experiments were conducted on different hardware platforms, including both CPU and GPU platforms. The running time and memory usage of the model were measured on these platforms. The specific hardware platforms used are as follows:

(1) CPU platform: Intel i7-12700K with 16 GB of RAM;
(2) GPU platforms: NVIDIA RTX 2060 with 8 GB of VRAM; NVIDIA RTX 3080 with 10 GB of VRAM.

The evaluation of computational efficiency on different hardware platforms is shown in Table 5.

When running on the CPU, the inference time of STDA-YOLOv8 increased by approximately 90 ms compared to the original YOLOv8. This is because STDA-YOLOv8 introduces more convolution operations (such as multi-scale expansion convolution and feature fusion modules), increasing the computational complexity. The memory usage also increased slightly, mainly because the CAM and the FRM need to store more intermediate features.

On the GPU platform, the increase in inference time of STDA-YOLOv8 compared to the original YOLOv8 was smaller. For the RTX 2060, the inference time increased by about 10 ms, while for the RTX 3080, the increase was about 8 ms. This indicates that the parallel acceleration of the GPU can effectively mitigate the consumption brought by additional convolution operations to a certain extent. The VRAM usage also increased slightly, but the increase was relatively small, indicating that the model optimization design achieved a good balance in VRAM usage.

In summary, although the improved STDA-YOLOv8 has a slight increase in inference time and memory usage, its detection accuracy and recall rate have significantly improved compared to the original model. Such a design makes a certain trade-off between accuracy and efficiency. Meanwhile, on high-performance GPU platforms (such as RTX 3080), STDA-YOLOv8 can maintain lower inference time and moderate VRAM usage, making it more applicable in practice, especially in tasks requiring high-precision detection of small targets.

## 5. Summary and Outlook

To address the issues of false positives and missed detections in small-target detection caused by network limitations and imbalanced training data, this paper proposes an improved YOLOv8 algorithm for small-target detection, named STDA-YOLOv8. This algorithm introduces a new small-target detection network structure into the YOLOv8 model, which combines a Contextual Augmentation Module (CAM) and a Feature Refinement Module (FRM). The CAM incorporates multi-scale expansion convolutions, with different dilation rates to capture contextual information from various receptive fields. The FRM performs adaptive feature fusion in both channel and spatial dimensions, eliminating conflicting information between features of different scales to prevent the features of small objects from being overwhelmed. Additionally, a new data augmentation method called Copy–Reduce–Paste is proposed, increasing the contribution of small objects to the loss function during training and ensuring balanced training. Ablation experiments on the VisDrone dataset and the PASCAL VOC dataset verified the effectiveness of the above improvements, and comparative experiments showed that the STDA-YOLOv8 algorithm outperformed mainstream algorithms in small-target detection.

The study by Ashraf [28] shows that there is degradation in the performance of the YOLO series models in bad weather environments, and thus how to improve the model performance through more advanced data enhancement techniques and more robust feature extraction modules will become an important research direction. Target detection in degraded imaging environments (e.g., bad weather, low light, etc.) will also be further explored. Efforts will also continue to improve the model’s network structure, such as introducing lightweight Transformers to simplify the model while maintaining detection performance or adding Convolutional Block Attention Modules to enhance recognition accuracy and efficiency. Furthermore, additional suitable data augmentation methods for small-target detection will be explored, such as adversarial data augmentation or methods based on generative adversarial networks, to address the issue of imbalanced training data.

## Figures and Tables

**Table 1:** Performance comparison table before and after data enhancement.

Dataset	Scene Type	Quantity	Quantity	Proportion	Proportion	P/%	P/%	R/%	R/%	${\mathrm{mAP}}_{0.5}$/%	${\mathrm{mAP}}_{0.5}$/%
		(Before)	(After)	(Before)	(After)	(Before)	(After)	(Before)	(After)	(Before)	(After)
VisDrone	Urban scene	200	400	1:5	1:2	77.6	82.1	83.3	85.3	88.2	89.6
VisDrone	Rural scene	150	300	1:4	1:2	79.8	84.2	84.2	86.8	89	90
VisDrone	Sparse scene	120	240	1:6	1:3	81	86.5	84.7	88.4	89.8	91.3
VisDrone	Crowded scene	180	360	1:5	1:2	80.2	85.9	83.5	87.6	90.5	91.9
PASCAL VOC	Universal scene	200	400	1:3	1:1.5	82.3	86.8	85.4	88.5	90	92.2
Overall average		850	1700	1:4.6	1:2.1	80.2	85.1	84.2	87.3	89.5	91

**Table 2:** Training parameter settings.

Parameter	Value
Epochs	600
Patience	30
Batch	8
Imgsz	8
Workers	4
Optimizer	SGD
Weight_decay	0.0005
Irf	0.05
Momentum	0.937
Warmuop_momentum	0.8
Close_mosaic	10
Patience	50

**Table 3:** Ablation study results.

YOLOv8	CAM	CAM	CAM	FRM	VisDrone	VisDrone	VisDrone	PASCAL VOC	PASCAL VOC	PASCAL VOC	Params/	${\mathrm{GFLOP}}_{s}$	Model Size/
	(1, 3, 5)	(2, 4, 6)	(1, 2, 3)		P/%	R/%	${\mathrm{mAP}}_{0.5}$/%	P/%	R/%	${\mathrm{mAP}}_{0.5}$/%	$10^{6}$		MB
√					77.6	83.3	88.2	81	84	88.5	3.1	8.1	6.2
√	√				82.1	85.3	89.6	85.5	87	90.3	2.5	7.4	6.3
√		√			83.5	87	90.2	86.8	87.5	91	2.6	7.6	6.4
√			√		84.2	86.8	90	86.5	87.2	91.2	2.5	7.5	6.4
√	√			√	85.9	88	91.3	87.9	88.5	92	2.6	7.6	6.3
√		√		√	87.2	89.2	92	88.5	89.5	92.8	2.6	7.5	6.3
√			√	√	87	88.9	91.8	88.2	89	92.5	2.5	7.4	6.2
√	√	√		√	88.6	91	92.8	89.5	90.5	93.2	2.6	7.5	6.2
√	√		√	√	88.3	90.7	92.5	89.1	90	93.3	2.6	7.5	6.2
√	√	√	√	√	89.6	93.8	93.5	90.3	92.5	94.2	2.6	7.5	6.1

**Table 4:** Results of comparative experiments.

Model (Model	VisDrone	VisDrone	VisDrone	PASCAL VOC	PASCAL VOC	PASCAL VOC	Params/$10^{6}$	${\mathrm{GFLOP}}_{s}$
Size: Small)	P/%	R/%	${\mathrm{mAP}}_{0.5}$/%	P/%	R/%	${\mathrm{mAP}}_{0.5}$/%		
YOLOv5	77.8	78.6	84.3	80.9	83.4	85.5	2.4	7.5
QueryDet	72.1	78.2	82.5	80.5	82.6	83.8	4.1	10.8
YOLOv7	76.8	83.2	85.6	80.7	83.1	85.8	5.7	11.3
YOLOv8	77.6	83.3	88.2	81	84	88.5	3.1	8.1
literatures [37]	80.5	85.2	85.7	82.3	85.4	88.9	4.3	9.7
literatures [38]	78.9	82.1	83.9	81.2	83.6	86.7	4.7	10.2
STDA-YOLOv8	89.6	93.8	93.5	90.3	92.5	94.2	2.6	7.5

**Table 5:** Computational efficiency evaluation.

Hardware Platform	Model	Average Reasoning Time (ms)	Memory Usage (MB)	GPU Video Memory Usage (MB)
Intel i7-12700K	YOLOv8	420	2900	
Intel i7-12700K	STDA-YOLOv8	510	3100	
NVIDIA RTX 2060	YOLOv8	38		2200
NVIDIA RTX 2060	STDA-YOLOv8	48		2600
NVIDIA RTX 3080	YOLOv8	21		1600
NVIDIA RTX 3080	STDA-YOLOv8	29		1800

## Data Availability

The original data presented in the study are openly available in VisDrone dataset at “https://github.com/VisDrone/VisDrone-Dataset, accessed on 13 March 2025” and PASCAL VOC dataset at “http://host.robots.ox.ac.uk/pascal/VOC/, accessed on 13 March 2025”.
